# Tuberostemoamide hemihydrate

**DOI:** 10.1107/S1600536811043340

**Published:** 2011-10-29

**Authors:** Rong-Rong Zhang, Zhi-Guo Ma, Guo-Qiang Li, Paul Pui-Hay But, Ren-Wang Jiang

**Affiliations:** a Guangdong Province Key Laboratory of Pharmacodynamic Constituents of, Traditional Chinese Medicine and New Drugs Research, Institute of Traditional Chinese Medicine and Natural Products, Jinan University, Guangzhou 510632, People’s Republic of China; b School of Biological Sciences, The Chinese University of Hong Kong, Hong Kong, People’s Republic of China

## Abstract

In the crystal structure of the title compound {systematic name: (1′*S*,2*R*,2′*R*,3′*S*,6′*R*)-3′-ethyl-4-methyl-5*H*-5′-oxa-10′-aza­spiro­[furan-2,4′-tricyclo­[8.3.0.0^2,6^]trideca­ne]-5,11′-dione hemihydrate}, C_17_H_23_NO_4_·0.5H_2_O, the asymmetric unit contains two mol­ecules of tuberostemoamide with similar conformations and one water mol­ecule. The tuberostemoamide mol­ecule is composed of one seven-membered ring (*A*) and three five-membered rings (*B*, *C* and *D*). Ring *A* exists in a chair conformation, both rings *B* and *C* exist in envelope conformations, and ring *D* is almost planar with a mean deviation of 0.0143 (4) Å in one molecule and 0.0095 (3) Å in the other.. The dihedral angles between the planes of rings *C* and *D* are 75.1 (3)° in one mol­ecule and 74.5 (3)° for the other. The solvent water mol­ecule links the tuberostemoamide mol­ecules through O—H⋯O(ketone) hydrogen bonds. Weak C—H⋯O inter­actions are also present, involving both the water mol­ecule and a heterocyclic ether O-atom acceptor.

## Related literature

For general background, see: Pilli & Ferreira de Oliveira (2000 ▶); Jiang *et al.* (2006 ▶). For the biological activity of Stemona alkaloids, see: Xu *et al.* (2010 ▶); Lin *et al.* (2008 ▶); Hu *et al.* (2009 ▶).
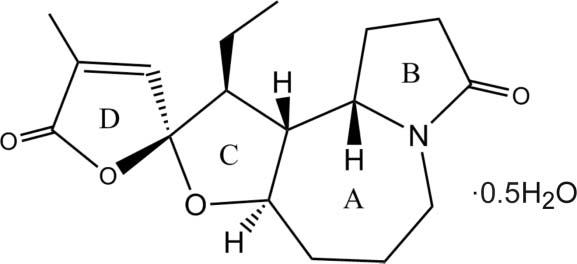


## Experimental

### 

#### Crystal data

C_17_H_23_NO_4_·0.5H_2_O
*M* *_r_* = 314.37
Orthorhombic, 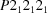

*a* = 8.6412 (2) Å
*b* = 10.7998 (2) Å
*c* = 36.1685 (7) Å
*V* = 3375.36 (12) Å^3^
*Z* = 8
Cu *K*α radiation
μ = 0.73 mm^−1^
*T* = 298 K
0.42 × 0.30 × 0.27 mm

#### Data collection

Oxford Diffraction Gemini S Ultra CCD diffractometer
Absorption correction: multi-scan (*SADABS*; Sheldrick, 2004 ▶) *T*_min_ = 0.822, *T*_max_ = 1.000
8573 measured reflections
4837 independent reflections
4514 reflections with *I* > 2*I*)
*R*_int_ = 0.021

#### Refinement

*R*[*F* ^2^ > 2σ(*F* ^2^)] = 0.050
*wR*(*F* ^2^) = 0.142
*S* = 1.06
4837 reflections
412 parameters
2 restraints
H atoms treated by a mixture of independent and constrained refinement
Δρ_max_ = 0.51 e Å^−3^
Δρ_min_ = −0.24 e Å^−3^
Absolute structure: Flack (1983 ▶), 1743 Friedel pairs
Flack parameter: −0.1 (2)

### 

Data collection: *SMART* (Bruker, 1998 ▶); cell refinement: *SAINT* (Bruker, 1998 ▶); data reduction: *SAINT*; program(s) used to solve structure: *SHELXS97* (Sheldrick, 2008 ▶); program(s) used to refine structure: *SHELXL97* (Sheldrick, 2008 ▶); molecular graphics: *XP* (Bruker, 1998 ▶); software used to prepare material for publication: *SHELXTL* (Sheldrick, 2008 ▶).

## Supplementary Material

Crystal structure: contains datablock(s) I, global. DOI: 10.1107/S1600536811043340/zs2152sup1.cif

Structure factors: contains datablock(s) I. DOI: 10.1107/S1600536811043340/zs2152Isup2.hkl

Additional supplementary materials:  crystallographic information; 3D view; checkCIF report

## Figures and Tables

**Table 1:** Hydrogen-bond geometry (Å, °)

*D*—H⋯*A*	*D*—H	H⋯*A*	*D*⋯*A*	*D*—H⋯*A*
O1*W*—H1*WA*⋯O1	0.81	1.98	2.796 (3)	175
O1*W*—H1*WB*⋯O1′	0.83	1.97	2.784 (3)	171
C5′—H5′*A*⋯O3^i^	0.97	2.58	3.545 (4)	178
C10—H10*A*⋯O1*W*^ii^	0.98	2.58	3.450 (4)	149

Symmetry codes: (i) 
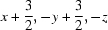
; (ii) 
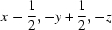
.

**Fig. 1.**
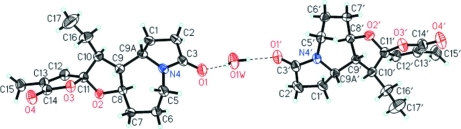
The molecular structure of the title compound showing 30% probability displacement ellipsoids and the atom-numbering scheme. Inter-species hydrogen bonds are shown as dashed lines.

**Fig. 2.**
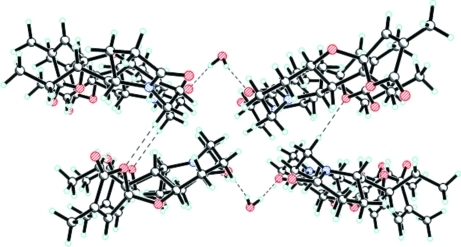
Packing diagram viewed down the a axis showing O—H···O and C—H···O hydrogen bonds as dashed lines.

## supplementary crystallographic information

### Comment

The title compound 2(C_17_H_23_NO_4_) . H_2_O (Fig. 1) is a hydrate of tuberostemoamide which has been isolated from the roots of Stemona tuberosa. This plant is a rich source of Stemona alkaloids (Pilli & Ferreira de Oliveira, 2000; Jiang *et al.*, 2006) with anti-tussive activity (Xu *et al.*, 2010). Although the phytochemical properties of tuberostemoamide have been studied Hu *et al.*, 2009); Lin *et al.*, 2008) the crystal structure has not previously been reported. In this study, we report the structure of tuberostemoamide hemihydrate.

The asymmetric unit contains two molecules of tuberostemoamide [(1) and (2)] with similar conformations and one water molecule (Fig. 1). The tuberostemoamide molecule is composed of one seven-membered ring (*A*) and three five-membered rings (*B*, *C* and *D*). Ring *A* exists in a chair conformation. Ring *B* exists in an envelope conformation with C1 displaced by 0.4695 Å from the least-squares plane of the remaining four atoms (C2, C3, N4 and C9*A*), and the corresponding value for molecule (2) is 0.4868 Å. Ring *C* also exhibits an envelope conformation with C10 displaced by 0.5930 Å from the least-squares plane of the remaining four atoms (C8, C8, O2 and C11), and the corresponding value for molecule (2) is 0.5886 Å. Ring *D* is planar with a mean deviation 0.0142 Å for (1) and 0.0101 Å for (2). The dihedral angles between the planes of the rings *C* and *D* in molecule (1) is 75.1 (3)° and 74.5 (3)° for molecule (2). The absolute configuration for the compound was not determined with certainty in this analysis.

The water molecule links the tuberostemoamide molecules through O—H···O(ketone) hydrogen bonds (Table 1) with only weak intermolecular C—H···O interactions present, involving both the water molecule and a hetero-ring ether O-acceptor (Fig. 2).

### Experimental

The dry ground herbal sample Radix stemonae (8.0 kg) was refluxed with 95% EtOH. After evaporation of the solvent, the crude extract was acidified with 4% HCl and filtered, and the filtrate was washed with diethyl ether (800 ml). The pH of the aqueous layer was raised to 9 with aqueous ammonia (35%) and then extracted with Et_2_O (800 ml). The Et_2_O layer was evaporated to afford the crude alkaloids (25 g), which was subjected to column chromatography over silica gel, and eluted with cyclohexane-ethyl acetate (10:1 to 0:1) to yield fourteen fractions. Fraction 8 (4 g) was subjected to reverse phase silica gel chromatography to yield seven subfractions, after which the second subfraction (60% CH_3_CN, 8 mg) was purified by preparative HPLC eluted by 25% CH_3_CN to yield tuberostemoamide (4 mg). The colorless crystals were obtained from a methanol solution at room temperature.

### Refinement

The C-bound H atoms were positioned geometrically and were included in the refinement in the riding-model approximation, with C—H = 0.96 Å (CH_3_) and *U*_iso_(H) = 1.5*U*_eq_(C); 0.97 Å (CH_2_) and *U*_iso_(H) = 1.2*U*_eq_(C); 0.93 Å (aryl H) and *U*_iso_(H)= 1.2*U*_eq_(C); O—H = 0.82 Å and *U*_iso_(H) = 1.5*U*_eq_(O). In the absence of a suitable heavy atom the Flack parameter determined for the parent compound [-0.1 (2) for 1743 Friedel pairs] is not definitive of the absolute configuration of tuberostemoamide but for the chosen enantiomer, the 5 chiral centers of both independent molecules are C8(*R*), C9(*R*), C9*A*(*S*), C10(*S*), C11(*R*).

### Crystal data

**Table:**

C_17_H_23_NO_4_·0.5H_2_O	*F*(000) = 1352
*M**_r_* = 314.37	*D*_x_ = 1.237 Mg m^−^^3^
Orthorhombic, *P*2_1_2_1_2_1_	Cu *K*α radiation, λ = 1.54184 Å
Hall symbol: P 2ac 2ab	Cell parameters from 4774 reflections
*a* = 8.6412 (2) Å	θ = 3.7–62.5°
*b* = 10.7998 (2) Å	µ = 0.73 mm^−^^1^
*c* = 36.1685 (7) Å	*T* = 298 K
*V* = 3375.36 (12) Å^3^	Block, colorless
*Z* = 8	0.42 × 0.30 × 0.27 mm

### Data collection

**Table:**

Oxford Diffraction Gemini S Ultra CCD diffractometer	4837 independent reflections
Radiation source: fine-focus sealed tube	4514 reflections with *I* > 2˘*I*)
graphite	*R*_int_ = 0.021
ω scans	θ_max_ = 62.6°, θ_min_ = 4.3°
Absorption correction: multi-scan (*SADABS*; Sheldrick, 2004)	*h* = −9→9
*T*_min_ = 0.822, *T*_max_ = 1.000	*k* = −12→10
8573 measured reflections	*l* = −19→41

### Refinement

**Table:**

Refinement on *F*^2^	Secondary atom site location: difference Fourier map
Least-squares matrix: full	Hydrogen site location: inferred from neighbouring sites
*R*[*F*^2^ > 2σ(*F*^2^)] = 0.050	H atoms treated by a mixture of independent and constrained refinement
*wR*(*F*^2^) = 0.142	*w* = 1/[σ^2^(*F*_o_^2^) + (0.0913*P*)^2^ + 0.4948*P*] where *P* = (*F*_o_^2^ + 2*F*_c_^2^)/3
*S* = 1.06	(Δ/σ)_max_ < 0.001
4837 reflections	Δρ_max_ = 0.51 e Å^−^^3^
412 parameters	Δρ_min_ = −0.24 e Å^−^^3^
2 restraints	Absolute structure: Flack (1983), 1743 Friedel pairs
Primary atom site location: structure-invariant direct methods	Flack parameter: −0.1 (2)

### Special details

**Table:**

Geometry. All e.s.d.'s (except the e.s.d. in the dihedral angle between two l.s. planes) are estimated using the full covariance matrix. The cell e.s.d.'s are taken into account individually in the estimation of e.s.d.'s in distances, angles and torsion angles; correlations between e.s.d.'s in cell parameters are only used when they are defined by crystal symmetry. An approximate (isotropic) treatment of cell e.s.d.'s is used for estimating e.s.d.'s involving l.s. planes.
Refinement. Refinement of *F*^2^ against ALL reflections. The weighted *R*-factor *wR* and goodness of fit *S* are based on *F*^2^, conventional *R*-factors *R* are based on *F*, with *F* set to zero for negative *F*^2^. The threshold expression of *F*^2^ > σ(*F*^2^) is used only for calculating *R*-factors(gt) *etc*. and is not relevant to the choice of reflections for refinement. *R*-factors based on *F*^2^ are statistically about twice as large as those based on *F*, and *R*- factors based on ALL data will be even larger.

### Fractional atomic coordinates and isotropic or equivalent isotropic displacement parameters (Å^2^)

**Table:**

	*x*	*y*	*z*	*U*_iso_*/*U*_eq_	
O1'	0.3987 (3)	0.6908 (2)	0.08626 (7)	0.0818 (7)	
O2'	0.9749 (2)	0.7450 (2)	0.18688 (6)	0.0688 (6)	
O3'	1.0512 (3)	0.9441 (2)	0.20168 (6)	0.0792 (7)	
O4'	1.2437 (4)	1.0530 (3)	0.22680 (11)	0.1210 (12)	
C1'	0.4852 (4)	0.7634 (3)	0.18058 (9)	0.0663 (8)	
H1'A	0.4397	0.8047	0.2018	0.080*	
H1'B	0.5442	0.6924	0.1890	0.080*	
C2'	0.3601 (4)	0.7249 (4)	0.15240 (10)	0.0828 (10)	
H2'A	0.3262	0.6405	0.1567	0.099*	
H2'B	0.2713	0.7797	0.1537	0.099*	
C3'	0.4403 (3)	0.7360 (3)	0.11593 (9)	0.0614 (7)	
N4'	0.5691 (3)	0.8034 (2)	0.12056 (6)	0.0530 (5)	
C5'	0.6762 (4)	0.8299 (3)	0.09050 (7)	0.0583 (7)	
H5'A	0.7141	0.9140	0.0931	0.070*	
H5'B	0.6214	0.8244	0.0672	0.070*	
C6'	0.8127 (4)	0.7416 (3)	0.08984 (7)	0.0617 (7)	
H6'A	0.7732	0.6575	0.0902	0.074*	
H6'B	0.8673	0.7527	0.0666	0.074*	
C7'	0.9266 (3)	0.7555 (3)	0.12079 (8)	0.0612 (7)	
H7'A	0.9796	0.8341	0.1181	0.073*	
H7'B	1.0035	0.6904	0.1188	0.073*	
C8'	0.8538 (3)	0.7500 (3)	0.15902 (7)	0.0513 (6)	
H8'A	0.7900	0.6752	0.1609	0.062*	
C9'	0.7566 (3)	0.8615 (2)	0.16928 (7)	0.0477 (6)	
H9'A	0.8006	0.9338	0.1567	0.057*	
C9A'	0.5859 (3)	0.8521 (2)	0.15822 (7)	0.0514 (6)	
H9AB	0.5397	0.9349	0.1592	0.062*	
C10'	0.7858 (4)	0.8767 (3)	0.21083 (7)	0.0547 (6)	
H10B	0.7249	0.8140	0.2239	0.066*	
C11'	0.9562 (4)	0.8396 (3)	0.21282 (8)	0.0596 (7)	
C12'	1.0220 (4)	0.8032 (3)	0.24871 (9)	0.0681 (8)	
H12B	0.9854	0.7383	0.2632	0.082*	
C13'	1.1403 (4)	0.8751 (4)	0.25765 (9)	0.0762 (9)	
C14'	1.1569 (4)	0.9670 (4)	0.22847 (11)	0.0810 (10)	
C15'	1.2413 (6)	0.8803 (6)	0.29114 (12)	0.1187 (17)	
H15D	1.2159	0.8129	0.3073	0.178*	
H15E	1.2251	0.9574	0.3038	0.178*	
H15F	1.3478	0.8739	0.2838	0.178*	
C16'	0.7445 (6)	1.0033 (4)	0.22668 (10)	0.0858 (11)	
H16C	0.8230	1.0618	0.2188	0.103*	
H16D	0.6473	1.0294	0.2158	0.103*	
C17'	0.7300 (10)	1.0123 (6)	0.26649 (16)	0.157 (3)	
H17D	0.7049	1.0960	0.2732	0.236*	
H17E	0.8260	0.9891	0.2779	0.236*	
H17F	0.6493	0.9579	0.2748	0.236*	
O1	−0.0168 (3)	0.5873 (2)	0.01640 (7)	0.0779 (6)	
O2	−0.5911 (2)	0.31108 (18)	−0.03973 (5)	0.0528 (4)	
O3	−0.6774 (2)	0.36455 (16)	−0.09836 (5)	0.0564 (5)	
O4	−0.8917 (3)	0.3499 (2)	−0.13237 (6)	0.0742 (6)	
C1	−0.1041 (3)	0.3420 (3)	−0.04581 (9)	0.0591 (7)	
H1A	−0.1618	0.2844	−0.0305	0.071*	
H1B	−0.0599	0.2974	−0.0665	0.071*	
C2	0.0210 (3)	0.4071 (3)	−0.02354 (9)	0.0595 (7)	
H2A	0.0564	0.3556	−0.0033	0.071*	
H2B	0.1087	0.4283	−0.0390	0.071*	
C3	−0.0582 (3)	0.5213 (3)	−0.00945 (8)	0.0545 (6)	
N4	−0.1863 (2)	0.5401 (2)	−0.02972 (6)	0.0515 (5)	
C5	−0.2939 (3)	0.6411 (3)	−0.02211 (9)	0.0610 (7)	
H5A	−0.3387	0.6697	−0.0452	0.073*	
H5B	−0.2378	0.7097	−0.0111	0.073*	
C6	−0.4218 (3)	0.6016 (3)	0.00366 (9)	0.0610 (7)	
H6A	−0.4763	0.6751	0.0120	0.073*	
H6B	−0.3755	0.5633	0.0252	0.073*	
C7	−0.5400 (3)	0.5116 (3)	−0.01270 (8)	0.0544 (7)	
H7A	−0.6114	0.4867	0.0066	0.065*	
H7B	−0.5992	0.5543	−0.0316	0.065*	
C8	−0.4694 (3)	0.3976 (2)	−0.02940 (7)	0.0437 (5)	
H8A	−0.4020	0.3583	−0.0111	0.052*	
C9	−0.3777 (3)	0.4177 (2)	−0.06508 (7)	0.0457 (6)	
H9A	−0.4245	0.4879	−0.0781	0.055*	
C9A	−0.2073 (3)	0.4480 (2)	−0.05906 (7)	0.0493 (6)	
H9AA	−0.1645	0.4812	−0.0821	0.059*	
C10	−0.4123 (3)	0.3013 (3)	−0.08746 (7)	0.0529 (6)	
H10A	−0.3504	0.2334	−0.0772	0.063*	
C11	−0.5799 (3)	0.2795 (2)	−0.07690 (7)	0.0500 (6)	
C12	−0.6566 (3)	0.1570 (2)	−0.08304 (8)	0.0576 (7)	
H12A	−0.6192	0.0819	−0.0742	0.069*	
C13	−0.7846 (3)	0.1697 (2)	−0.10266 (7)	0.0536 (6)	
C14	−0.7969 (3)	0.3011 (2)	−0.11322 (7)	0.0523 (6)	
C15	−0.9043 (4)	0.0787 (3)	−0.11504 (10)	0.0691 (8)	
H15A	−0.8783	−0.0022	−0.1059	0.104*	
H15B	−0.9079	0.0770	−0.1416	0.104*	
H15C	−1.0036	0.1028	−0.1056	0.104*	
C16	−0.3768 (5)	0.3105 (4)	−0.12896 (9)	0.0858 (11)	
H16A	−0.4557	0.3617	−0.1404	0.103*	
H16B	−0.2787	0.3530	−0.1319	0.103*	
C17	−0.3690 (9)	0.1966 (7)	−0.14874 (13)	0.158 (3)	
H17A	−0.3458	0.2129	−0.1742	0.238*	
H17B	−0.4666	0.1546	−0.1470	0.238*	
H17C	−0.2892	0.1455	−0.1383	0.238*	
O1W	0.1914 (4)	0.5014 (3)	0.06972 (10)	0.1025 (9)	
H1WA	0.128 (6)	0.529 (6)	0.0552 (14)	0.154*	
H1WB	0.256 (6)	0.553 (5)	0.0765 (16)	0.154*	

### Atomic displacement parameters (Å^2^)

**Table:**

	*U*^11^	*U*^22^	*U*^33^	*U*^12^	*U*^13^	*U*^23^
O1'	0.0742 (14)	0.0875 (15)	0.0837 (14)	−0.0204 (13)	−0.0242 (12)	−0.0017 (13)
O2'	0.0611 (12)	0.0800 (13)	0.0653 (11)	0.0199 (11)	−0.0105 (10)	−0.0137 (10)
O3'	0.0698 (14)	0.0857 (15)	0.0821 (14)	−0.0206 (13)	−0.0149 (12)	0.0174 (13)
O4'	0.091 (2)	0.112 (2)	0.160 (3)	−0.042 (2)	−0.032 (2)	0.014 (2)
C1'	0.0494 (15)	0.082 (2)	0.0676 (16)	−0.0068 (16)	0.0064 (14)	0.0036 (15)
C2'	0.0488 (17)	0.102 (3)	0.098 (2)	−0.0145 (18)	0.0042 (17)	0.005 (2)
C3'	0.0522 (15)	0.0579 (16)	0.0743 (18)	−0.0020 (14)	−0.0125 (15)	0.0050 (14)
N4'	0.0514 (12)	0.0490 (11)	0.0584 (12)	−0.0085 (10)	−0.0059 (10)	0.0023 (10)
C5'	0.0723 (18)	0.0559 (15)	0.0468 (13)	−0.0160 (15)	−0.0060 (13)	0.0078 (11)
C6'	0.0733 (19)	0.0614 (16)	0.0504 (13)	−0.0130 (16)	0.0116 (14)	−0.0043 (12)
C7'	0.0525 (15)	0.0713 (17)	0.0600 (15)	−0.0030 (14)	0.0116 (14)	−0.0109 (14)
C8'	0.0498 (14)	0.0514 (13)	0.0527 (13)	0.0004 (12)	−0.0008 (12)	−0.0050 (11)
C9'	0.0488 (14)	0.0443 (13)	0.0499 (12)	−0.0011 (11)	0.0021 (11)	−0.0011 (10)
C9A'	0.0452 (14)	0.0500 (14)	0.0590 (13)	0.0032 (12)	−0.0023 (11)	−0.0015 (12)
C10'	0.0597 (16)	0.0550 (15)	0.0495 (13)	−0.0005 (14)	0.0021 (12)	−0.0034 (12)
C11'	0.0627 (17)	0.0599 (17)	0.0562 (14)	−0.0039 (15)	−0.0038 (13)	−0.0001 (13)
C12'	0.078 (2)	0.0655 (18)	0.0606 (15)	−0.0022 (17)	−0.0118 (15)	0.0012 (14)
C13'	0.077 (2)	0.081 (2)	0.0708 (18)	−0.0006 (19)	−0.0223 (17)	−0.0044 (17)
C14'	0.063 (2)	0.083 (2)	0.097 (3)	−0.0072 (19)	−0.0125 (18)	−0.006 (2)
C15'	0.115 (4)	0.149 (4)	0.092 (3)	−0.008 (3)	−0.049 (3)	−0.011 (3)
C16'	0.113 (3)	0.071 (2)	0.073 (2)	0.017 (2)	0.002 (2)	−0.0231 (16)
C17'	0.199 (7)	0.147 (5)	0.126 (4)	0.035 (5)	0.007 (5)	−0.050 (4)
O1	0.0652 (13)	0.0750 (14)	0.0937 (15)	−0.0013 (12)	−0.0213 (12)	−0.0129 (12)
O2	0.0478 (10)	0.0594 (10)	0.0513 (9)	−0.0123 (9)	0.0013 (8)	−0.0019 (8)
O3	0.0622 (11)	0.0405 (9)	0.0666 (10)	−0.0009 (9)	−0.0147 (9)	0.0045 (8)
O4	0.0718 (13)	0.0615 (12)	0.0894 (14)	−0.0005 (11)	−0.0272 (12)	0.0171 (11)
C1	0.0472 (14)	0.0535 (15)	0.0766 (17)	0.0057 (13)	0.0086 (14)	−0.0014 (13)
C2	0.0387 (13)	0.0603 (16)	0.0796 (17)	0.0042 (12)	0.0029 (13)	0.0114 (14)
C3	0.0432 (13)	0.0489 (14)	0.0715 (16)	−0.0047 (12)	−0.0008 (13)	0.0056 (13)
N4	0.0423 (11)	0.0430 (11)	0.0694 (13)	0.0004 (10)	−0.0003 (10)	0.0047 (10)
C5	0.0508 (14)	0.0412 (13)	0.0910 (19)	0.0048 (12)	−0.0096 (15)	−0.0014 (13)
C6	0.0551 (16)	0.0517 (15)	0.0762 (17)	0.0089 (13)	−0.0022 (14)	−0.0142 (14)
C7	0.0451 (14)	0.0561 (15)	0.0621 (15)	0.0054 (12)	0.0043 (12)	−0.0048 (12)
C8	0.0390 (12)	0.0449 (12)	0.0471 (12)	−0.0019 (11)	−0.0027 (10)	0.0023 (10)
C9	0.0458 (12)	0.0465 (13)	0.0449 (12)	0.0005 (11)	0.0003 (11)	0.0059 (10)
C9A	0.0465 (13)	0.0511 (14)	0.0504 (13)	−0.0016 (12)	0.0086 (11)	0.0050 (11)
C10	0.0575 (15)	0.0511 (13)	0.0501 (13)	0.0047 (13)	0.0009 (12)	−0.0020 (11)
C11	0.0543 (15)	0.0437 (13)	0.0520 (13)	0.0039 (12)	−0.0072 (12)	0.0008 (11)
C12	0.0657 (17)	0.0416 (13)	0.0656 (15)	0.0039 (13)	−0.0101 (14)	0.0035 (12)
C13	0.0589 (15)	0.0397 (13)	0.0621 (14)	0.0011 (12)	−0.0061 (13)	−0.0011 (11)
C14	0.0556 (15)	0.0461 (13)	0.0553 (13)	0.0025 (13)	−0.0057 (13)	0.0019 (11)
C15	0.0646 (18)	0.0528 (16)	0.090 (2)	−0.0069 (15)	−0.0156 (17)	−0.0028 (15)
C16	0.102 (3)	0.097 (3)	0.0583 (17)	0.010 (2)	0.0197 (19)	−0.0043 (17)
C17	0.192 (6)	0.202 (6)	0.081 (3)	0.027 (6)	0.009 (4)	−0.047 (4)
O1W	0.104 (2)	0.0833 (18)	0.120 (2)	−0.0337 (17)	−0.0342 (18)	0.0232 (15)

### Geometric parameters (Å, °)

**Table:**

O1'—C3'	1.233 (4)	O2—C11	1.390 (3)
O2'—C11'	1.396 (4)	O2—C8	1.455 (3)
O2'—C8'	1.454 (3)	O3—C14	1.351 (3)
O3'—C14'	1.354 (4)	O3—C11	1.468 (3)
O3'—C11'	1.453 (4)	O4—C14	1.195 (3)
O4'—C14'	1.195 (5)	C1—C2	1.521 (4)
C1'—C9A'	1.525 (4)	C1—C9A	1.528 (4)
C1'—C2'	1.542 (5)	C1—H1A	0.9700
C1'—H1'A	0.9700	C1—H1B	0.9700
C1'—H1'B	0.9700	C2—C3	1.499 (4)
C2'—C3'	1.495 (5)	C2—H2A	0.9700
C2'—H2'A	0.9700	C2—H2B	0.9700
C2'—H2'B	0.9700	C3—N4	1.343 (4)
C3'—N4'	1.340 (4)	N4—C5	1.460 (3)
N4'—C5'	1.456 (4)	N4—C9A	1.466 (3)
N4'—C9A'	1.467 (3)	C5—C6	1.507 (4)
C5'—C6'	1.517 (4)	C5—H5A	0.9700
C5'—H5'A	0.9700	C5—H5B	0.9700
C5'—H5'B	0.9700	C6—C7	1.529 (4)
C6'—C7'	1.498 (4)	C6—H6A	0.9700
C6'—H6'A	0.9700	C6—H6B	0.9700
C6'—H6'B	0.9700	C7—C8	1.501 (4)
C7'—C8'	1.520 (4)	C7—H7A	0.9700
C7'—H7'A	0.9700	C7—H7B	0.9700
C7'—H7'B	0.9700	C8—C9	1.530 (3)
C8'—C9'	1.515 (4)	C8—H8A	0.9800
C8'—H8'A	0.9800	C9—C10	1.524 (4)
C9'—C9A'	1.532 (4)	C9—C9A	1.524 (4)
C9'—C10'	1.533 (3)	C9—H9A	0.9800
C9'—H9'A	0.9800	C9A—H9AA	0.9800
C9A'—H9AB	0.9800	C10—C11	1.517 (4)
C10'—C16'	1.525 (4)	C10—C16	1.535 (4)
C10'—C11'	1.528 (4)	C10—H10A	0.9800
C10'—H10B	0.9800	C11—C12	1.496 (4)
C11'—C12'	1.470 (4)	C12—C13	1.321 (4)
C12'—C13'	1.324 (5)	C12—H12A	0.9300
C12'—H12B	0.9300	C13—C14	1.473 (4)
C13'—C14'	1.456 (5)	C13—C15	1.496 (4)
C13'—C15'	1.494 (5)	C15—H15A	0.9600
C15'—H15D	0.9600	C15—H15B	0.9600
C15'—H15E	0.9600	C15—H15C	0.9600
C15'—H15F	0.9600	C16—C17	1.425 (7)
C16'—C17'	1.449 (6)	C16—H16A	0.9700
C16'—H16C	0.9700	C16—H16B	0.9700
C16'—H16D	0.9700	C17—H17A	0.9600
C17'—H17D	0.9600	C17—H17B	0.9600
C17'—H17E	0.9600	C17—H17C	0.9600
C17'—H17F	0.9600	O1W—H1WA	0.81 (2)
O1—C3	1.229 (4)	O1W—H1WB	0.82 (2)
			
C11'—O2'—C8'	110.8 (2)	C14—O3—C11	109.35 (19)
C14'—O3'—C11'	108.9 (3)	C2—C1—C9A	103.5 (2)
C9A'—C1'—C2'	102.6 (3)	C2—C1—H1A	111.1
C9A'—C1'—H1'A	111.2	C9A—C1—H1A	111.1
C2'—C1'—H1'A	111.2	C2—C1—H1B	111.1
C9A'—C1'—H1'B	111.2	C9A—C1—H1B	111.1
C2'—C1'—H1'B	111.2	H1A—C1—H1B	109.0
H1'A—C1'—H1'B	109.2	C3—C2—C1	103.6 (2)
C3'—C2'—C1'	103.7 (2)	C3—C2—H2A	111.0
C3'—C2'—H2'A	111.0	C1—C2—H2A	111.0
C1'—C2'—H2'A	111.0	C3—C2—H2B	111.0
C3'—C2'—H2'B	111.0	C1—C2—H2B	111.0
C1'—C2'—H2'B	111.0	H2A—C2—H2B	109.0
H2'A—C2'—H2'B	109.0	O1—C3—N4	124.6 (3)
O1'—C3'—N4'	124.5 (3)	O1—C3—C2	127.0 (3)
O1'—C3'—C2'	126.9 (3)	N4—C3—C2	108.4 (2)
N4'—C3'—C2'	108.6 (3)	C3—N4—C5	122.3 (2)
C3'—N4'—C5'	122.8 (2)	C3—N4—C9A	113.2 (2)
C3'—N4'—C9A'	113.1 (2)	C5—N4—C9A	124.4 (2)
C5'—N4'—C9A'	124.0 (2)	N4—C5—C6	111.9 (2)
N4'—C5'—C6'	112.5 (2)	N4—C5—H5A	109.2
N4'—C5'—H5'A	109.1	C6—C5—H5A	109.2
C6'—C5'—H5'A	109.1	N4—C5—H5B	109.2
N4'—C5'—H5'B	109.1	C6—C5—H5B	109.2
C6'—C5'—H5'B	109.1	H5A—C5—H5B	107.9
H5'A—C5'—H5'B	107.8	C5—C6—C7	115.5 (2)
C7'—C6'—C5'	115.9 (2)	C5—C6—H6A	108.4
C7'—C6'—H6'A	108.3	C7—C6—H6A	108.4
C5'—C6'—H6'A	108.3	C5—C6—H6B	108.4
C7'—C6'—H6'B	108.3	C7—C6—H6B	108.4
C5'—C6'—H6'B	108.3	H6A—C6—H6B	107.5
H6'A—C6'—H6'B	107.4	C8—C7—C6	113.9 (2)
C6'—C7'—C8'	113.8 (2)	C8—C7—H7A	108.8
C6'—C7'—H7'A	108.8	C6—C7—H7A	108.8
C8'—C7'—H7'A	108.8	C8—C7—H7B	108.8
C6'—C7'—H7'B	108.8	C6—C7—H7B	108.8
C8'—C7'—H7'B	108.8	H7A—C7—H7B	107.7
H7'A—C7'—H7'B	107.7	O2—C8—C7	109.7 (2)
O2'—C8'—C9'	105.0 (2)	O2—C8—C9	104.40 (18)
O2'—C8'—C7'	109.5 (2)	C7—C8—C9	115.7 (2)
C9'—C8'—C7'	114.9 (2)	O2—C8—H8A	108.9
O2'—C8'—H8'A	109.1	C7—C8—H8A	108.9
C9'—C8'—H8'A	109.1	C9—C8—H8A	108.9
C7'—C8'—H8'A	109.1	C10—C9—C9A	116.2 (2)
C8'—C9'—C9A'	114.6 (2)	C10—C9—C8	103.3 (2)
C8'—C9'—C10'	103.5 (2)	C9A—C9—C8	114.2 (2)
C9A'—C9'—C10'	114.9 (2)	C10—C9—H9A	107.5
C8'—C9'—H9'A	107.8	C9A—C9—H9A	107.5
C9A'—C9'—H9'A	107.8	C8—C9—H9A	107.5
C10'—C9'—H9'A	107.8	N4—C9A—C9	111.6 (2)
N4'—C9A'—C1'	102.2 (2)	N4—C9A—C1	102.1 (2)
N4'—C9A'—C9'	111.2 (2)	C9—C9A—C1	116.6 (2)
C1'—C9A'—C9'	116.9 (2)	N4—C9A—H9AA	108.7
N4'—C9A'—H9AB	108.7	C9—C9A—H9AA	108.7
C1'—C9A'—H9AB	108.7	C1—C9A—H9AA	108.7
C9'—C9A'—H9AB	108.7	C11—C10—C9	100.5 (2)
C16'—C10'—C11'	116.3 (3)	C11—C10—C16	116.5 (3)
C16'—C10'—C9'	115.2 (3)	C9—C10—C16	115.3 (3)
C11'—C10'—C9'	100.2 (2)	C11—C10—H10A	108.0
C16'—C10'—H10B	108.2	C9—C10—H10A	108.0
C11'—C10'—H10B	108.2	C16—C10—H10A	108.0
C9'—C10'—H10B	108.2	O2—C11—O3	108.5 (2)
O2'—C11'—O3'	108.5 (2)	O2—C11—C12	109.3 (2)
O2'—C11'—C12'	110.7 (3)	O3—C11—C12	102.7 (2)
O3'—C11'—C12'	103.6 (2)	O2—C11—C10	105.8 (2)
O2'—C11'—C10'	105.7 (2)	O3—C11—C10	108.5 (2)
O3'—C11'—C10'	109.1 (2)	C12—C11—C10	121.6 (2)
C12'—C11'—C10'	119.0 (3)	C13—C12—C11	111.0 (2)
C13'—C12'—C11'	110.9 (3)	C13—C12—H12A	124.5
C13'—C12'—H12B	124.5	C11—C12—H12A	124.5
C11'—C12'—H12B	124.5	C12—C13—C14	107.5 (2)
C12'—C13'—C14'	107.4 (3)	C12—C13—C15	132.2 (3)
C12'—C13'—C15'	132.1 (4)	C14—C13—C15	120.4 (2)
C14'—C13'—C15'	120.3 (4)	O4—C14—O3	122.0 (2)
O4'—C14'—O3'	121.9 (4)	O4—C14—C13	128.7 (3)
O4'—C14'—C13'	128.9 (4)	O3—C14—C13	109.3 (2)
O3'—C14'—C13'	109.1 (3)	C13—C15—H15A	109.5
C13'—C15'—H15D	109.5	C13—C15—H15B	109.5
C13'—C15'—H15E	109.5	H15A—C15—H15B	109.5
H15D—C15'—H15E	109.5	C13—C15—H15C	109.5
C13'—C15'—H15F	109.5	H15A—C15—H15C	109.5
H15D—C15'—H15F	109.5	H15B—C15—H15C	109.5
H15E—C15'—H15F	109.5	C17—C16—C10	116.4 (4)
C17'—C16'—C10'	117.0 (4)	C17—C16—H16A	108.2
C17'—C16'—H16C	108.0	C10—C16—H16A	108.2
C10'—C16'—H16C	108.0	C17—C16—H16B	108.2
C17'—C16'—H16D	108.0	C10—C16—H16B	108.2
C10'—C16'—H16D	108.0	H16A—C16—H16B	107.3
H16C—C16'—H16D	107.3	C16—C17—H17A	109.5
C16'—C17'—H17D	109.5	C16—C17—H17B	109.5
C16'—C17'—H17E	109.5	H17A—C17—H17B	109.5
H17D—C17'—H17E	109.5	C16—C17—H17C	109.5
C16'—C17'—H17F	109.5	H17A—C17—H17C	109.5
H17D—C17'—H17F	109.5	H17B—C17—H17C	109.5
H17E—C17'—H17F	109.5	H1WA—O1W—H1WB	113 (6)
C11—O2—C8	110.84 (19)		
			
C9A'—C1'—C2'—C3'	28.3 (4)	C9A—C1—C2—C3	28.4 (3)
C1'—C2'—C3'—O1'	163.4 (3)	C1—C2—C3—O1	161.7 (3)
C1'—C2'—C3'—N4'	−15.8 (4)	C1—C2—C3—N4	−17.6 (3)
O1'—C3'—N4'—C5'	−0.5 (5)	O1—C3—N4—C5	−2.0 (4)
C2'—C3'—N4'—C5'	178.8 (3)	C2—C3—N4—C5	177.3 (2)
O1'—C3'—N4'—C9A'	176.5 (3)	O1—C3—N4—C9A	179.5 (3)
C2'—C3'—N4'—C9A'	−4.2 (3)	C2—C3—N4—C9A	−1.3 (3)
C3'—N4'—C5'—C6'	−97.1 (3)	C3—N4—C5—C6	−91.8 (3)
C9A'—N4'—C5'—C6'	86.2 (3)	C9A—N4—C5—C6	86.6 (3)
N4'—C5'—C6'—C7'	−69.8 (3)	N4—C5—C6—C7	−70.3 (3)
C5'—C6'—C7'—C8'	53.7 (4)	C5—C6—C7—C8	53.6 (3)
C11'—O2'—C8'—C9'	1.6 (3)	C11—O2—C8—C7	124.5 (2)
C11'—O2'—C8'—C7'	125.5 (3)	C11—O2—C8—C9	0.0 (3)
C6'—C7'—C8'—O2'	171.3 (3)	C6—C7—C8—O2	172.6 (2)
C6'—C7'—C8'—C9'	−70.9 (3)	C6—C7—C8—C9	−69.7 (3)
O2'—C8'—C9'—C9A'	−150.5 (2)	O2—C8—C9—C10	−23.5 (2)
C7'—C8'—C9'—C9A'	89.2 (3)	C7—C8—C9—C10	−144.1 (2)
O2'—C8'—C9'—C10'	−24.5 (3)	O2—C8—C9—C9A	−150.7 (2)
C7'—C8'—C9'—C10'	−144.9 (2)	C7—C8—C9—C9A	88.7 (3)
C3'—N4'—C9A'—C1'	22.5 (3)	C3—N4—C9A—C9	144.6 (2)
C5'—N4'—C9A'—C1'	−160.5 (2)	C5—N4—C9A—C9	−33.9 (3)
C3'—N4'—C9A'—C9'	147.9 (2)	C3—N4—C9A—C1	19.3 (3)
C5'—N4'—C9A'—C9'	−35.1 (3)	C5—N4—C9A—C1	−159.2 (2)
C2'—C1'—C9A'—N4'	−30.1 (3)	C10—C9—C9A—N4	−164.9 (2)
C2'—C1'—C9A'—C9'	−151.7 (3)	C8—C9—C9A—N4	−44.8 (3)
C8'—C9'—C9A'—N4'	−44.1 (3)	C10—C9—C9A—C1	−48.2 (3)
C10'—C9'—C9A'—N4'	−163.9 (2)	C8—C9—C9A—C1	72.0 (3)
C8'—C9'—C9A'—C1'	72.6 (3)	C2—C1—C9A—N4	−28.6 (3)
C10'—C9'—C9A'—C1'	−47.2 (3)	C2—C1—C9A—C9	−150.5 (2)
C8'—C9'—C10'—C16'	161.7 (3)	C9A—C9—C10—C11	162.2 (2)
C9A'—C9'—C10'—C16'	−72.5 (4)	C8—C9—C10—C11	36.3 (2)
C8'—C9'—C10'—C11'	36.2 (3)	C9A—C9—C10—C16	−71.6 (3)
C9A'—C9'—C10'—C11'	161.9 (2)	C8—C9—C10—C16	162.5 (3)
C8'—O2'—C11'—O3'	−94.7 (3)	C8—O2—C11—O3	−92.3 (2)
C8'—O2'—C11'—C12'	152.3 (3)	C8—O2—C11—C12	156.4 (2)
C8'—O2'—C11'—C10'	22.2 (3)	C8—O2—C11—C10	24.0 (3)
C14'—O3'—C11'—O2'	−118.9 (3)	C14—O3—C11—O2	−113.1 (2)
C14'—O3'—C11'—C12'	−1.3 (4)	C14—O3—C11—C12	2.5 (3)
C14'—O3'—C11'—C10'	126.3 (3)	C14—O3—C11—C10	132.4 (2)
C16'—C10'—C11'—O2'	−160.9 (3)	C9—C10—C11—O2	−37.4 (2)
C9'—C10'—C11'—O2'	−36.0 (3)	C16—C10—C11—O2	−162.7 (3)
C16'—C10'—C11'—O3'	−44.4 (3)	C9—C10—C11—O3	78.8 (2)
C9'—C10'—C11'—O3'	80.5 (3)	C16—C10—C11—O3	−46.4 (3)
C16'—C10'—C11'—C12'	74.0 (4)	C9—C10—C11—C12	−162.6 (2)
C9'—C10'—C11'—C12'	−161.1 (3)	C16—C10—C11—C12	72.2 (4)
O2'—C11'—C12'—C13'	115.6 (3)	O2—C11—C12—C13	111.1 (3)
O3'—C11'—C12'—C13'	−0.4 (4)	O3—C11—C12—C13	−4.0 (3)
C10'—C11'—C12'—C13'	−121.7 (3)	C10—C11—C12—C13	−125.3 (3)
C11'—C12'—C13'—C14'	1.9 (4)	C11—C12—C13—C14	3.8 (3)
C11'—C12'—C13'—C15'	177.3 (4)	C11—C12—C13—C15	−177.6 (3)
C11'—O3'—C14'—O4'	−175.9 (4)	C11—O3—C14—O4	−179.0 (3)
C11'—O3'—C14'—C13'	2.5 (4)	C11—O3—C14—C13	−0.5 (3)
C12'—C13'—C14'—O4'	175.5 (5)	C12—C13—C14—O4	176.3 (3)
C15'—C13'—C14'—O4'	−0.6 (7)	C15—C13—C14—O4	−2.5 (5)
C12'—C13'—C14'—O3'	−2.7 (4)	C12—C13—C14—O3	−2.1 (3)
C15'—C13'—C14'—O3'	−178.8 (4)	C15—C13—C14—O3	179.1 (2)
C11'—C10'—C16'—C17'	−79.4 (6)	C11—C10—C16—C17	−78.4 (5)
C9'—C10'—C16'—C17'	163.8 (5)	C9—C10—C16—C17	164.2 (5)

### Hydrogen-bond geometry (Å, °)

**Table:**

*D*—H···*A*	*D*—H	H···*A*	*D*···*A*	*D*—H···*A*
O1W—H1WA···O1	0.81	1.98	2.796 (3)	175.
O1W—H1WB···O1'	0.83	1.97	2.784 (3)	171.
C5'—H5'A···O3^i^	0.97	2.58	3.545 (4)	178.
C10—H10A···O1W^ii^	0.98	2.58	3.450 (4)	149.

Symmetry codes: (i) *x*+3/2, −*y*+3/2, −*z*; (ii) *x*−1/2, −*y*+1/2, −*z*.
